# Reduced resting-state functional connectivity between insula and inferior frontal gyrus and superior temporal gyrus in hoarding disorder

**DOI:** 10.3389/fpsyt.2024.1399062

**Published:** 2024-06-19

**Authors:** Kenta Kato, Hirofumi Tomiyama, Keitaro Murayama, Taro Mizobe, Akira Matsuo, Nami Nishida, Kou Matukuma, Mingi Kang, Kenta Sashikata, Kazufumi Kikuchi, Osamu Togao, Tomohiro Nakao

**Affiliations:** ^1^ Department of Neuropsychiatry, Graduate School of Medical Sciences, Kyushu University, Fukuoka, Japan; ^2^ Department of Neuropsychiatry, Kyushu University Hospital, Fukuoka, Japan; ^3^ Graduate School of Human Environment Studies, Kyushu University, Fukuoka, Japan; ^4^ Department of Clinical Radiology, Graduate School of Medical Sciences, Kyushu University, Fukuoka, Japan

**Keywords:** hoarding disorder, resting-state, functional connectivity, seed-based analysis, cognitive control

## Abstract

**Background:**

Hoarding disorder (HD) is characterized by cognitive control impairments and abnormal brain activity in the insula and anterior cingulate cortex (ACC) during disposal of personal items or certain executive function tasks. However, whether there are any changes in resting-state functional connectivity of the insula and ACC remains unclear.

**Methods:**

A total of 55 subjects, including 24 patients with HD and 31 healthy controls (HCs), participated in the study. We acquired resting-state functional magnetic resonance imaging data and examined group differences in functional connectivity from the insula and ACC in whole-brain voxels.

**Results:**

In patients with HD, functional connectivity was significantly lower between the right insula and right inferior frontal gyrus (IFG) and left superior temporal gyrus (STG) compared to HCs. There was no correlation between these connectivities and HD symptoms.

**Conclusions:**

Although the clinical implication is uncertain, our results suggest that patients with HD have resting-state functional alterations between the insula and IFG and STG, corresponding with the results of previous fMRI studies. These findings provide new insight into the neurobiological basis of HD.

## 1 Introduction

Hoarding disorder (HD) is a mental disorder characterized by difficulty disposing of one’s own possessions, regardless of their actual value. HD was previously considered a subtype of obsessive-compulsive disorder (OCD) but is now considered a distinct disorder in the Diagnostic and Statistical Manual of Mental Disorders fifth edition (DSM-5) (1). According to a meta-analysis of epidemiological studies, the prevalence of HD is estimated at approximately 2.5% (2), higher than OCD at 1–2% (3). However, many individuals with HD do not receive help because of the lack of treatment methods and medical interventions as well as their poor insight into their hoarding behavior and its impact on their living condition (4). Moreover, the neurobiological features of HD remain unclear.

Previous studies have shown that patients with HD could have impairments in some domains of cognitive control function, including response inhibition, working memory, attention, memory, categorization (5–8), emotional dysregulation such as intolerance of uncertainty (9, 10), and biased decision-making patterns such as inefficient choices to maximize value (11, 12). Task functional magnetic resonance imaging (fMRI) studies using a go/no-go task, stop-signal task, or switch-signal task have shown abnormal brain activity in a wide range of brain regions, including the orbitofrontal cortex, prefrontal cortex, insula, anterior cingulate cortex (ACC), precentral gyrus, fusiform cortex, visual cortex, striatum, and cerebellum, compared to healthy controls or patients with OCD, although the results vary among studies (13–15). Compared to patients with OCD and healthy individuals, patients with HD showed greater brain activity of the right insula when making decisions about personal possessions and lower activity in the ACC and left insula when making decisions about another person’s possessions (16). Similar results have been reported previously (17). Stevens et al. evaluated brain activity in patients with HD and controls during acquisition and discarding of objects, as hoarding symptoms, and a semantic processing task as a nonrelated executive function (17). They showed that there was greater brain activity in the insula, cingulate gyrus, and inferior frontal gyrus (IFG) during the acquisition and discarding tasks than during the semantic processing task in the HD group compared with the non-HD group. Recent fMRI studies examining the effect of cognitive behavioral therapy in patients with HD have shown abnormal brain activity in several regions, including the insula and ACC, during simulated decisions on whether to keep or discard objects (18). These studies indicate that HD is associated with neurophysiological abnormalities and abnormal activation of the insula and ACC during both executive control and decision-making tasks. However, it is unclear whether the altered activity of the insula and ACC exists during the resting state, and not only during cognitive tasks.

Resting-state functional MRI (rsfMRI) is a method to explore the neurophysiological basis of resting-state functional connectivity (rsFC), which is defined as the temporal correlation of blood-oxygen-level-dependent (BOLD) signals between spatially distinct brain regions at rest (19). The advantages of rsfMRI are that there is no need to concurrently administer and monitor tasks, and task performance does not affect the interpretation of the results. Although the biological background of rsfMRI is not fully understood compared with that of task-related fMRI, rsfMRI has the advantages of being performed in patients who are unable to perform tasks accurately and has a low burden of data acquisition. These characteristics allow for broad sampling of the patient population. In other psychiatric disorders, mega-analyses using a large amount of rsfMRI data have been conducted (20, 21). Many studies have focused on resting-state functional connectivity and reported abnormalities that may lead to the discovery of biomarkers related to psychiatric disorders (21–25). Up to date, there is only one study focusing rsfMRI in HD, and that study using independent component analysis (ICA) (26). ICA is a data-driven approach that can assess general connectivity patterns in the entire brain without prior hypotheses. In their experiment, they measured the resting-state brain activity in patients with HD, HD and major depressive disorder (MDD), MDD, and healthy subjects. The participants were separated into two groups according to the presence or absence of an HD diagnosis. They found that participants with HD diagnosis have shown abnormalities in major brain networks, including the salience network (SN), default mode network (DMN), and frontoparietal network (FPN) compared to participants without HD diagnosis. However, there were no common abnormal networks in the HD and HD+MDD groups. These results may have been influenced by the small sample size in each group (8 in the MDD group and 10 in the HD+MDD group), and the fact that the non-HD group included some patients with MDD. It remains unclear whether there are any alterations in resting-state functional connectivity of the insula and ACC. Seed-based analysis can test hypotheses focusing specific brain areas by measuring the functional connectivity between specific regions of interest (ROIs) and the whole brain (27), and the interpretation of the results is straightforward compared with the ICA approach (28). The aim of this study was to clarify the rsFC, using seed-based analysis with ROIs, related to insula and ACC, which may be involved in the neurobiological basis of HD. The aim of this study was to clarify the rsFC, using seed-based analysis with ROIs, related to insula and ACC, which may be involved in the neurobiological basis of HD. Based on previous literature on HD, we hypothesized that patients with HD may have abnormalities in the rsFC of the insula and ACC. Specifically, the IFG, orbitofrontal cortex, dorsolateral prefrontal cortex, and superior temporal gyrus (STG) are anatomically connected to the insula and ACC and have shown abnormalities along with the insula and ACC in previous task-related fMRI on HD. Therefore, we expected that patients with HD may show abnormalities in the rsFC between the insula and ACC and these regions.

## 2 Methods

### 2.1 Participants

A total of 56 subjects, including 25 patients with HD and 31 healthy controls (HCs) matched for age, sex, and dominant hand, were enrolled. One patient with HD was excluded because of a high rate of invalid volumes, as described below. A total of 24 patients with HD and 31 HCs were included in this study. All patients were recruited from the Department of Neuropsychiatry at Kyushu University Hospital, Japan. HCs were recruited from the local community and interviewed using the Structured Clinical Interview for DSM-IV non-patient edition (SCID-I/NP) (29). None of the HCs had Axis I disorders. Participants were excluded if they had a neurological disorder, head injury, serious medical conditions, a history of drug or alcohol dependence, or comorbidities of major psychiatric disorders such as schizophrenia or bipolar disorder. All participants provided written informed consent before enrollment in the study. This study was conducted in accordance with the Declaration of Helsinki and approved by the Ethics Committee of Kyushu University.

### 2.2 Clinical assessments

All participants were evaluated for OCD severity, anxiety, and depression symptoms using the Yale-Brown Obsessive-Compulsive Scale (Y-BOCS) (30, 31), Hamilton Rating Scale for Anxiety (HAM-A), and Hamilton Rating Scale for Depression (HAM-D:17-item version) (32, 33). In the HD group, the Structured Interview for Hoarding Disorders (SIHD) (34) was used to confirm they fulfilled the DSM-5 criteria for HD as the primary diagnosis and to exclude secondary hoarding symptoms to OCD and autism spectrum disorder. The HD group was further evaluated for the severity of hoarding symptoms using the Hoarding Rating Scale-Interview (HRS-I) (35), Clutter Imaging Rating (CIR) (36), and Saving Inventory-Revised (SI-R) (37). Current comorbid disorders were also evaluated, including attention deficit hyperactivity disorder (ADHD), using the Structured Clinical Interview for DSM-IV Axis I Disorders-Patient Edition (SCID-I/P) (38) and Conners’ Adult ADHD Diagnostic Interview for DSM-IV (CAADID) (39). In addition, the severity of ADHD symptoms was evaluated using the Conners’ Adult ADHD Rating Scales-Self-Report: Long Version (CAARS-S:L) (40).

### 2.3 Brain image acquisition

Brain imaging was performed using a 3.0-Tesla MRI scanner (Achieva TX,Phillips Healthcare,Best, Netherlands) at the Department of Neuropsychiatry, Kyushu University Hospital, Japan. All the participants underwent fMRI sequencing. Prior to the resting-state fMRI scan, the participants were instructed to relax and keep their eyes opened and fixed on a gray cross symbol on the screen. First, we acquired a T2*-weighted gradient-echo echo-planar imaging (EPI) sequence (echo time [TE], 30 ms; repetition time [TR], 2500 ms; field of view [FOV], 212×212 mm; matrix, 64×64; slice thickness=3.2 mm; flip angle, 80°) from each participant. After an initial 10-s dummy scan, 240 real scans during a 10-min period were performed. High-resolution T1-weighted anatomical images (TE=3.8 ms; TR=8.2 ms; FOV 240×240 mm; flip angle 8°; slice thickness, 1 mm; inversion time=1026 ms) were also acquired for each participant after the EPI sequence.

### 2.4 MRI data preprocessing

The preprocessing steps were the same as those described in our previous studies (41, 42). CONN Toolbox 17.f (http://www.nitrc.org/projects/conn) was used for MRI data preprocessing. After removal of the first four volumes, 236 volumes were preprocessed using default preprocessing parameters of the CONN toolbox. Anatomical and functional images were realigned and normalized according to the standard Montreal Neurological Institute (MNI) template with slice timing corrected according to the slice order. Spatial smoothing was conducted with a 6-mm full-width-at-half-maximum Gaussian kernel. For temporal filtering, the fMRI data were band-pass filtered using the default settings in CONN (0.008–0.09 Hz). Twelve potential noise components, including six rigid-body parameters, were estimated for each participant. Outlier volumes were detected using Artifact Detection Tools (ART) software with a setting to detect volumes that had greater head motion than 0.9 mm from the previous image, and a z-score of 5 for scan-to-scan changes in the global signal. Participants with a high rate of outlier volume (>30%) were excluded from the analysis (43). There was no significant difference between HD and HC groups in motion parameters (mean FD [*t* =-0.384, *p >*0.05]).

### 2.5 MRI data analysis

After preprocessing, BOLD signal time-series correlations between each source pair for each participant were calculated over the resting time series, and Fisher z-transformation was applied. Seed-based connectivity maps were generated for each seed ROI of each participant. We created the seed ROIs as an 8 mm radius sphere with MNI coordinates in the right insula (30, 15, –3), left insula (-36, 12, -3), and ACC (0, 30, 21) based on a prior task-related functional MRI study of HD (16). Covariates included age, dummy-coded sex, and dummy-coded psychological medication use. We examined differences in functional connectivity from seed ROIs to whole-brain voxels between the HD and HC groups using a two-sample t-test. The significance level was set at individual voxel *p* < 0.001, and a cluster size threshold of *p* < 0.05 was corrected for false discovery rate. Furthermore, within the HD group, we investigated the correlations between the rsFC changes detected in group comparisons and the clinical symptoms. For detail, from the results of analysis of covariance group comparison between groups, we extracted the beta values, and transformed them to z-values, and then we conducted a correlation analysis between these altered rsFC and some clinical scores (HRS-I SI-R Total, SI-R Discarding, SI-R Clutter, SI-R Acquiring, CIR Mean, Y-BOCS, HAM-A, HAM-D, and CAARS-S:L).

## 3 Results

### 3.1 Demographic and clinical characteristics

The characteristics of patients with HD and HCs are shown in **Table 1**. The groups did not differ significantly in age (*t* (53) = 0.0244, *p* = 0.98 > 0.05), sex (χ2 (1) =0.017, *p* = 0.99 > 0.05), or handedness (χ2 (1) =0.293, *p* = 0.98 > 0.05). The HD group had hoarding symptoms above the cutoff value of the SI-R, and the severity of these symptoms was moderate or higher in the HRS-I. The HD group had significantly higher Y-BOCS (*t* (53) = 11.1, *p* < 0.001), HAM-D (*t* (53) = 4.18, *p* < 0.001), and HAM-A (*t* (53) = 3.85, *p* < 0.001) scores than HCs. Six participants in the HD group were diagnosed with HD only, and 18 participants had a variety of comorbidities, including major depressive disorder (n = 4), OCD (n = 8), ADHD (n = 10), post-traumatic stress disorder (PTSD) (n = 3), and anxiety disorder (n = 5), including specific phobia (n = 3), social anxiety disorder (n= 2), panic disorder (n = 1), and generalized anxiety disorder (n = 2). According to the SIHD, SCID-I/P, and CAADID, all participants in the HD group met the criteria for primary HD and did not show hoarding symptoms secondary to comorbidities. Twelve patients in the HD group were psychotropic medication-free, whereas the other 12 were taking antidepressants (n = 10), major tranquilizers (n = 1), non-stimulant ADHD medications (n = 3), and other medications. None of the participants in the HC group had taken psychotropic drugs during their lifetime.

**Table 1:** Demographic and clinical characteristics.

	Patients with HD (N=24)	Healthy control (N=31)	p value
**Age (years)**	42 ± 12.8	42 ± 12.3	0.98
**Female sex**	15 (62.5%)	20 (64.5%)	0.88^a^
**Right-handedness**	23 (95.8%)	30 (96.8%)	0.87^a^
Clinical assessments			
HRS-I	28.3 ± 7.11	–	–
SI-R Total	65.9 ± 12.7	–	–
SI-R Excessive Acquisition	15.9 ± 5.50	–	–
SI-R Difficulty Discarding	22.0 ± 4.48	–	–
SI-R Clutter	28 ± 6.35	–	–
CIR	4.38 ± 1.91	–	–
CAARS	22.2 ± 10.2	–	–
Y-BOCS	20.1 ± 8.88	0	<0.001
HAM-D	6.38 ± 6.91	0.42 ± 1.15	<0.001
HAM-A	6.17 ± 7.14	0.48 ± 1.23	<0.001
Comorbidities			
None	6 (25%)	31 (100%)	–
Major depression	4 (16.7%)	–	–
OCD	8 (33.3%)	–	–
ADHD	10 (41.7%)	–	–
Anxiety disorder	5 (20.8%)	–	–
PTSD	3 (12.5%)	–	–
Medication			
Medication-free	12 (50%)	31 (100%)	–
Antidepressants	10 (41.7%)	–	–
Major tranquilizers	1 (4.2%)	–	–
Nonstimulant ADHD drugs	3 (12.5%)	–	–
Non-GABA hypnotic drugs	2 (8.3%)	–	–
Benzodiazepines	6 (25%)	–	–
Mood stabilizers	1 (4.2%)	–	–

Clinical assessments: HRS-I, Hoarding Rating Scale-Interview; CIR, Clutter Imaging Rating; SI-R, Saving Inventory-Revised; CAARS, The Conners’ Adult ADHD Rating Scales-Self-Report; Y-BOCS, The Yale-Brown Obsessive-Compulsive Scale; HAM-D, Hamilton Rating Scale for Depression; HAM-A, Hamilton Rating Scale for Anxiety. Comorbidities: OCD, obsessive-compulsive disorder; ADHD, Attention Deficit Hyperactivity Disorder; PTSD, post-traumatic stress disorder. – means not available (N/A).

a χ^2^ test.

### 3.2 Between-group differences in resting-state functional connectivity from ROI to whole brain voxels between HD and HCs

**Figure 1:**
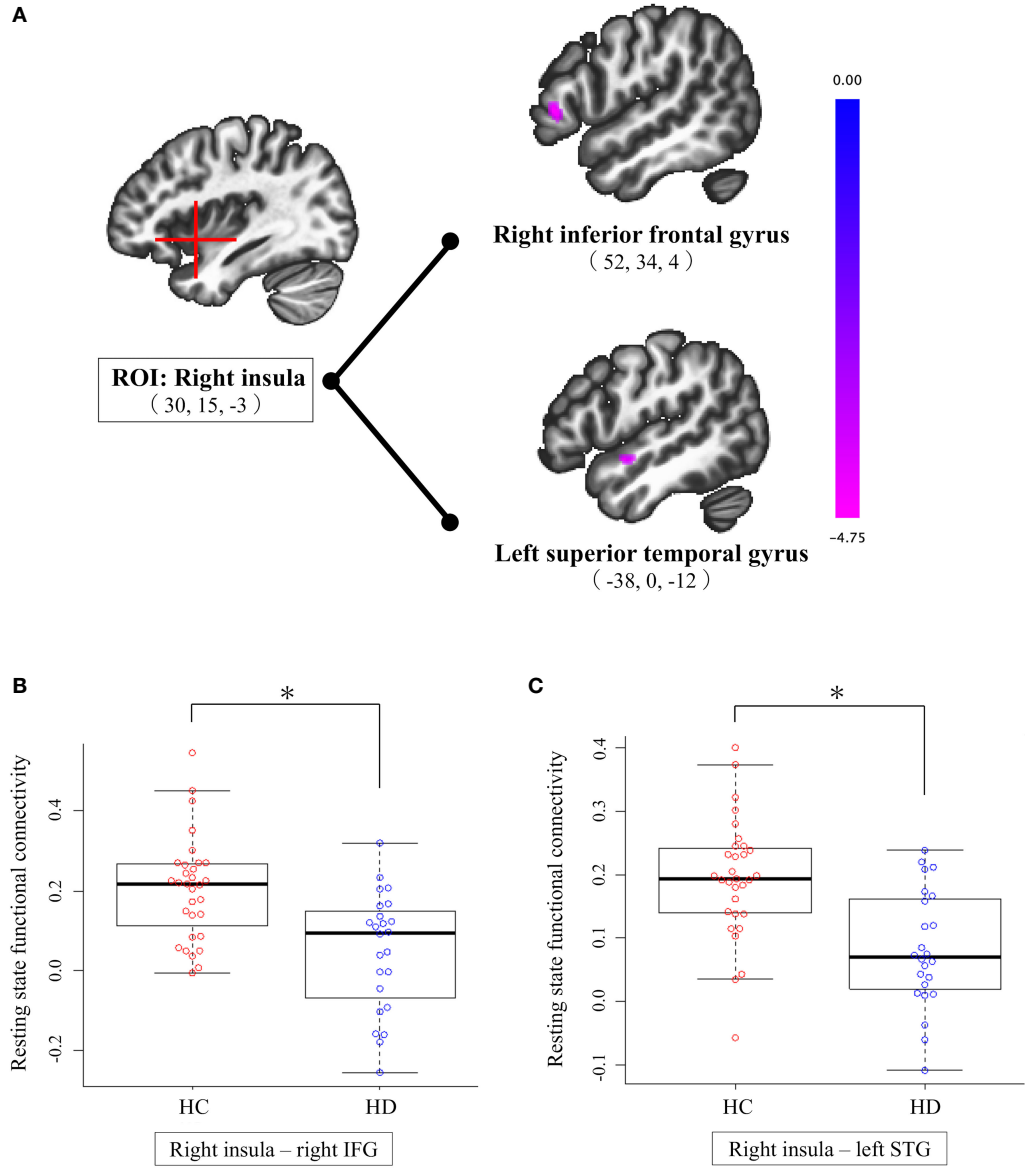
Results of seed-based analysis comparing right insula-right IFG and right insula-left STG connectivity between individuals with HD and HCs. **(A)** Patients with HD showed significantly lower functional connectivity from the right insula to the right IFG and left STG compared to HCs (cluster size corrected significance *p* < 0.05, after applying a per-voxel height threshold of *p* < 0.001). Peak coordinates are given in MNI space. **(B)** Group difference in right insula-right IFG connectivity; compared to HCs, individuals with HD exhibit significantly lower connectivity between right insula and right IFG. **(C)** Group difference in right insula-left STG connectivity; compared to HCs, individuals with HD exhibit significantly lower connectivity between right insula and left STG. HC, healthy control; HD, hoarding disorder; IFG, inferior frontal gyrus; MNI, Montreal Neurological Institute; ROI, region of interest; STG, superior temporal gyrus. *p-FDR <.05 (representing a Holm-corrected p-value adjusted for three ROIs comparisons after cluster-level FDR correction).

Connectivity comparisons between the groups revealed that patients with HD had significantly lower functional connectivity between the right insula and right IFG (peak MNI coordinates [52, 34, 4]) and between the right insula and left STG (peak MNI coordinates [-38, 0, -12]) compared to HCs (**Table 2**, **Figure 1**). There was no significant group difference in functional connectivity of the ACC and left insula in whole-brain voxels. When we included FD as a covariate of no interest, no changes were observed in our results. Furthermore, functional connectivity, which was abnormal in the comparison between groups, did not correlate significantly with the clinical assessments of the correlation coefficient (*p <*0.05) (**Supplementary Table 1**).

**Table 2:** Resting-state functional connectivity difference between groups.

Seed	Region	Ke	x	y	z	Direction	p-FDR ^a^
R insula	R inferior frontal gyrus	104	52	34	4	HD < HCs	0.044*
	L superior temporal gyrus	172	-38	0	-12	HD < HCs	0.0108*
L insula	–	–	–	–	–	–	–
ACC	–	–	–	–	–	–	–

*p-FDR <.05 (representing a Holm-corrected p-value adjusted for three ROIs comparisons after cluster-level FDR correction). Peak coordinates are given in MNI space.

ACC, anterior cingulate cortex; FDR, false discovery rate; HCs, healthy controls; HD, hoarding disorder; Ke, cluster extent; L, left; MNI, Montreal Neurological Institute; R, right; ROI, region of interest. – means there was no significant group difference in functional connectivity of the left insula and ACC.

aCluster size corrected p <.05 FDR after applying a voxel height threshold of p <.001.

## 4 Discussion

We found that patients with HD had lower rsFC between the right insula and right IFG and left STG compared to HCs. Given the lack of correlations between rsFC abnormalities and clinical symptoms of HD, the clinical implications of the present findings are not clear. However, considering the functional roles of these regions and previous findings on HD, we speculated that lower functional connectivity between the right insula and right IFG could be associated with the pathophysiology of HD. The right insula is known to identify salient stimuli from a variety of stimuli, including emotional responses, and facilitate information processing (44, 45). The IFG plays an important role in cognitive control (46–48), particularly relevant to emotional control through reappraisal strategies that regulate emotions by changing the interpretation of situations, stimuli, and mental states (49, 50). The IFG and insula are implicated in various cognitive control functions (45, 49–53), and functional connectivity between these regions has also been suggested to be related to emotional regulation (51, 52). Lower functional connectivity between the right insula and right IFG in the present study show an important overlap with those of previous findings on HD. In a study of HD for the disposal task, which we referenced in our seed ROIs, in addition to biphasic changes in brain activity in the insula and ACC, the activation of the insula and IFG in patients with HD was positively correlated with the degree of indecisiveness when considering the disposal of their own possessions (16). Another fMRI study using a disposal task suggested that patients with HD have semantic processing impairments based on the lower brain activity of the IFG during semantic processing tasks (17). Indecisiveness and semantic processing impairments can lead to hoarding symptoms, as they make it more difficult to make decisions about the disposal of their own possessions. It is hypothesized that changes in brain activity of the insula and IFG during hoarding symptom-induced tasks is related to the index of cognitive control in the context of indecisiveness and semantic processing in HD. Therefore, lower rsFC between the right insula and right IFG in the present study indicates that there are functional alterations in these regions, even in the resting state. As we did not perform neuropsychological tests of cognitive control function in this study, it is not possible to associate these results directly with impaired cognitive control in HD. However, it is interesting that regions identified in our study overlapped with the brain areas identified in previous task-related fMRI studies on HD. Alterations in brain regions that occur both during cognitive tasks and in the resting state may provide important insights into the neurobiological basis of HD.

In the present study, patients with HD had lower functional connectivity between the right insula and left STG compared to HCs. The abnormalities we observed in the STG match the results of several previous task-related fMRI studies on HD. Specifically, when patients with HD decided to keep or dispose of their personal possessions instead of the experimenter’s possessions, the brain activity in the left STG was positively correlated with self-reported sadness and “not just right” feelings (16). In another fMRI study, the brain activity in the left STG during both decisions to acquire and to discard possessions was significantly correlated with the severity of difficulty in discarding (17). Also, atrophy of the left temporal lobe, including the STG and insula, was associated with the prevalence of hoarding symptoms in frontotemporal dementia, particularly semantic dementia (54). The STG is hypothesized to be involved in semantic processing, underlying various cognitive control functions (46, 55–57). Previous studies showed that the STG is associated with the neurobiological basis of HD or hoarding symptoms. Our observation of lower rsFC between the right insula and left STG adds new evidence that patients with HD have abnormalities in the STG even in the resting state. However, because there was no correlation between the resting state functional change and the clinical symptoms, we cannot propose clinical applications of our results. Future studies are needed to examine the relationship between these rsFC changes in the STG and clinical implications for HD.

In contrast to our prior hypothesis, there was no significant difference in functional connectivity from the ACC between patients with HD and HCs, although previous neuroimaging studies reported characteristic ACC activities in patients with HD (14, 16, 17). The ACC and insula are key components of the salience network which regulates cognitive function and facilitates information processing (44, 45). Moreover, the ACC can be anatomically divided into dorsal and ventral regions which play different roles (Dixon et al., 2017, Etkin et al., 2011, Heilbronner and Hayden, 2016, Kolling et al., 2016, Lockwood and Wittmann, 2018). The dorsal ACC has been suggested to exhibit a central role in integrating information about errors, conflicts, negative feedback, and rewards, and assigning the appropriate cognitive control needed for action and decision making (53, 58, 59). The ventral ACC has been associated with the appraisal of interoceptive sensations related to subjective emotions (53, 60, 61). Previous neuroimaging studies on HD have shown that most ACC abnormalities are located in the dorsal regions which are often hyperactive during symptom-inducing tasks and hypoactive during non-symptom-inducing tasks (14, 16, 17). These abnormalities in the dorsal ACC may prevent patients with HD from correctly assessing the value of stimuli and may cause their abnormally high estimates of the risk of making errors, which lead to hoarding symptoms. Therefore, we selected an ROI in the dorsal part of the ACC and expected patients with HD to show abnormalities in the dorsal ACC and insula, even at rest. However, the present results suggest that rsFC changes in patients with HD are not engaging the dorsal ACC. While the interpretation of this discrepancy of the results is unclear, it should be noted that our study evaluated resting-state functional connectivity from ACC, not the regional brain activity of ACC itself. Moreover, owing to the following limitations of this study, further studies are needed to draw definite conclusions about the abnormalities of the ACC in patients with HD.

This study had several limitations. First, because of the small sample size and large number of comorbidities, it was not possible to analyze group differences according to comorbidity. In addition, the HD group had a high rate of psychotropic medication use because of comorbidities. HD is known to have a high comorbidity rate of approximately 75% (62), and the HD group in this study also had a similar rate of comorbidity. As a result, psychotropic drugs were used more frequently in the HD group. To examine the influence of comorbidities in the present study, we evaluated the correlation coefficient between the severity of comorbidities and abnormalities of functional connectivity. However, there was no significant correlation between altered functional connectivity and comorbidity severity. To test the effect of medication, we divided HD group into those with and without medication, and we investigated group difference from each ROIs using age and gender as covariate of no interest. As a result, there were no significant group differences between the group under medication and without medication. Second, we did not evaluate any cognitive functions in this study; therefore, we cannot predict the clinical implications of altered functional connectivity in HD. To assess whether the present findings are related to impairments in cognitive control in patients with HD, it is necessary to investigate the relationship between neuropsychological testing for cognitive control and brain activity during tasks or in the resting state. Future studies should increase the sample size and adjust groups according to the typical comorbidities of HD, such as depression, ADHD, and OCD, to test whether abnormal rsFC is a specific feature of HD. Third, methodological issues could have affected our results. In this study, we used seed-based analysis from the insula and ACC to whole-brain voxels. We selected the insula and ACC as ROIs with reference to a previous study on HD using symptom provocation paradigm task fMRI (16); therefore, we may have missed abnormalities in other regions that we did not evaluate. Furthermore, we determined the ROI coordinates based on that of previous fMRI studies, so a subtle difference in ROI coordinates could partially affect our results. Despite these limitations, this study has certain significance. It is the first to demonstrate that abnormalities related to the insula, which have been observed in task-related fMRI studies on HD, may also be present in the resting state.

## 5 Conclusion

We found lower rsFC between the right insula and right IFG and left STG in patients with HD compared to HCs. These brain regions overlap with those identified in previous task-related fMRI studies on HD. These results suggest that patients with HD have functional alterations in these brain areas, even in the resting state. Future studies are needed to clarify the clinical implication of these changes in resting-state functional connectivity in patients with HD.

## Data availability statement

The original contributions presented in the study are included in the article/**Supplementary Material**. Further inquiries can be directed to the corresponding author.

## Ethics statement

The studies involving humans were approved by The Ethics Committee of Kyushu University. The studies were conducted in accordance with the local legislation and institutional requirements. The participants provided their written informed consent to participate in this study.

## Author contributions

KK: Conceptualization, Formal analysis, Investigation, Visualization, Writing – original draft, Writing – review & editing. HT: Formal analysis, Funding acquisition, Methodology, Writing – review & editing. KeM: Funding acquisition, Writing – review & editing. TM: Investigation, Writing – review & editing. AM: Investigation, Writing – review & editing. NN: Investigation, Writing – review & editing. KoM: Investigation, Writing – review & editing. MK: Data curation, Writing – review & editing. KS: Data curation, Writing – review & editing. KfK: Investigation, Writing – review & editing. OT: Investigation, Writing – review & editing. TN: Funding acquisition, Supervision, Writing – review & editing.
