# Supplementary material for: Reduced resting-state functional connectivity between insula and inferior frontal gyrus and superior temporal gyrus in hoarding disorder
Source: Front Psychiatry. 2024 Jun 19;15:1399062. doi: 10.3389/fpsyt.2024.1399062 (PMC11223522; doi:10.3389/fpsyt.2024.1399062)
Supplement: Supplementary file 1 [file Table_1.docx]

| **Supplemental Table 1** The correlation between group differences in resting-state functional connectivity and clinical assessments in hoarding disorder group | | | |
| --- | --- | --- | --- |
| **Between-group differences in resting-state functional connectivity** | **Clinical Assessments** | **correlation coefficient** | ***p* value *** |
| Hypoconnectivity  between the right insula and right inferior frontal gyrus | HRS-I | -0.046 | 0.83 |
|  | SI-R Total | 0.033 | 0.88 |
|  | SI-R Discarding | 0.25 | 0.24 |
|  | SI-R Clutter | 0.21 | 0.31 |
|  | SIR-Acquiring | -0.3 | 0.15 |
|  | CIR | 0.05 | 0.81 |
|  | Y-BOCS | -0.03 | 0.89 |
|  | HAM-D | 0.05 | 0.81 |
|  | HAM-A | 0.15 | 0.5 |
|  | CAARS | 0.27 | 0.2 |
| Hypoconnectivity  between the right insula and left superior temporal gyrus | HRS-I | -0.17 | 0.43 |
|  | SI-R Total | 0.23 | 0.16 |
|  | SI-R Discarding | 0.39 | 0.056 |
|  | SI-R Clutter | 0.22 | 0.29 |
|  | SIR-Acquiring | -0.045 | 0.83 |
|  | CIR | 0.2 | 0.36 |
|  | Y-BOCS | 0.13 | 0.56 |
|  | HAM-D | 0.23 | 0.28 |
|  | HAM-A | 0.38 | 0.067 |
|  | CAARS | 0.23 | 0.27 |
| **Note** Abbreviations: HRS-I, Hoarding Rating Scale-Interview; CIR, Clutter Imaging Rating ; SI-R, Saving Inventory-Revised; CAARS, the Conners’ Adult ADHD Rating Scales-Self-Report; Y-BOCS, The Yale-Brown Obsessive-Compulsive Scale; HAM-D, Hamilton Rating Scale for Depression; HAM-A, Hamilton Rating Scale for Anxiety; HD, hoarding disorder; OCD, obsessive-compulsive disorder; ADHD, Attention Deficit Hyperactivity Disorder; PTSD, post-traumatic stress disorder. *p＜0.05 | | | |
|  |  |  |  |
|  |  |  |  |
|  |  |  |  |
